# Implementation of Outpatient Automated Stewardship Information System (OASIS^©^) audit and feedback in 2 healthcare systems

**DOI:** 10.1017/ash.2024.412

**Published:** 2024-10-28

**Authors:** Christine E. MacBrayne, Amy Keith, Nicole M. Poole, Cory Hussain, Joshua Tucker, Theresa Morin, Timothy C. Jenkins, Holly M. Frost

**Affiliations:** 1 Department of Pharmacy, Children’s Hospital Colorado, Aurora, CO, USA; 2 Center for Health Systems Research, Denver Health and Hospital Authority, Denver, CO, USA; 3 Department of Pediatrics, Division of Pediatric Infectious Diseases and Epidemiology, University of Colorado School of Medicine, Children’s Hospital Colorado, Aurora, CO, USA; 4 Division of Infectious Diseases, Denver Health and Hospital Authority, Denver, Colorado, USA; 5 Department of Analytics Resource Center, Children’s Hospital Colorado, Aurora, CO, USA; 6 Department of Pediatrics, Denver Health and Hospital Authority, Denver, CO, USA; 7 Department of Pediatrics, University of Colorado School of Medicine, Aurora, CO, USA

## Introduction

Providing feedback to clinicians on their prescribing over time and compared to their peers has been highly effective in reducing unnecessary antibiotic prescribing.^1^ However, obtaining data in the outpatient setting is resource intensive and often cost prohibitive.^2^ Outpatient Automated Stewardship Information System (OASIS^©^) was developed to alleviate these barriers.^2,3^ OASIS^©^ has been shown to increase guideline-concordant durations of therapy for acute otitis media (AOM).^3^ It uses common statistical software (R, R Foundation for Statistical Computing, Vienna, Austria) to abstract data from the electronic health record (EHR), analyze, and email feedback reports to clinicians. The code is free, and open-source and allows for automation;^4^ thus, continuous investment is minimal, and organizations need only invest resources in the initial setup. In this project, we aimed to assess the adaptations needed to implement OASIS^©^ across 2 healthcare systems for 4 respiratory metrics and, secondarily, assess the proportion of clinicians that viewed the reports and whether there were changes in prescribing associated with OASIS^©^ implementation.

## Methods

OASIS^©^ was implemented at Denver Health and Hospital Authority (DHHA) and Children’s Hospital Colorado (CHCO) from July 2022 to October 2023. Both organizations use Epic^®^ EHR (Verona, WI) but have different versions and builds. The intervention included the generation of individualized audit and feedback reports for clinicians that displayed their prescribing over time and compared to their peers. Targeted metrics included (1) antibiotics prescribed for acute respiratory tract infections (ARTI),^5–7^ (2) antibiotics prescribed for ARTIs for which antibiotics are never indicated,^6,7^ (3) first-line antibiotic therapy for AOM,^8^ and (4) 5-day durations of therapy for children 2 years of age and older with AOM.^3^

The primary outcome was adaptations needed to implement OASIS^©^. An evaluation utilizing the Framework for Reporting Adaptations and Modifications to Evidence-based Implementation Strategies (FRAME-IS) was conducted^9^ (Table 1). Secondary outcomes included fidelity, time to set up and maintain the program, barriers and facilitators to implementation, and changes in antibiotic prescribing for each metric (Supplemental Methods).

**Table 1.** Adaptations needed for OASIS^©^ implementation using the FRAME-IS framework

Adaptation name	Where?	When?	Why?	Who?
*Oasis code adaptations*				
Pointing code to different data elements at different organizations to generate reports.	Both sites	Pre-implementation	The code was adjusted by each site to pull the appropriate data elements from the EHR^1^ into the reports.	Data analyst
Did not display *n* for encounters <10	CHCO^2^	Pre-implementation	Compliance regulations at CHCO prohibited the display of the exact number of encounters if <10.	Data analyst
Identifying email addresses of report recipients	CHCO	Pre-implementation	EHR^1^ contained inaccurate or personal email addresses for some clinicians.	Stewards and data analysts
Sites opted to run R on an individual computer v R server which prevented automation. Each report was manually sent by simply re-running the program each month which automatically updated results.	Both sites	Pre-implementation	Sites did not have an R server license or did not have access to their R server license.	Data analysts
Identifying where to find the duration of therapy and which values to remove.	Both sites	Pre-implementation	Duration needed to be abstracted from the sig field. Prescribed amount could not be used to reliably calculate duration.	Stewards and data analysts
Making sure clinicians could see data for each clinic if they attended in multiple clinics.	Both sites	Pre-implementation	To provide a comprehensive view of prescribing patterns, the code was amended to ensure that if a clinician attended in multiple participating clinics within a reporting window, the report would include data from all practice locations.	Data analysts and clinicians
*Email adaptations*				
Creating OASIS-specific email for each organization.	Both sites	Pre-implementation	Identify issues with implementation, support users to make reports easy to access and read, track read receipts, and keep all user requests in a central location.	Stewards or data analysts
Tracking read receipts for fidelity monitoring	Both sites	Implementation/scale-up/maintenance	Upon clinician review of the report, a read receipt was sent to the OASIS email address to allow for tracking of report readership and patterns over time. These were tracked to monitor fidelity	Steward (CHCO^2^), research staff (DHHA^3^)
Organization-specific emails forwarded to personal emails.	CHCO^2^	Pre-implementation	While identifying the email addresses of the appropriate report recipients, we found that some clinicians had personal email addresses associated with their EHR^1^ accounts. This was corrected so that reports were sent only to institutional email addresses.	Data analyst
*Formatting adaptations*				
Reducing educational text to increase readability	Both sites	Scale-up	Report recipients recommended decreasing the amount of text displayed within the reports to ensure that the key takeaways were not being overlooked.	Stewards
Including denominator on reports for each metric (if value >10)	Both sites	Maintenance	Clinicians requested a denominator be added to reports for each metric.	Stewards and data analysts

1 Electronic health record.

2 Children’s Hospital of Colorado.

3 Denver Health and Hospital Authority.

This project was reviewed and approved by the Organizational Research Risk and Quality Improvement Review Panel at CHCO and the Quality Improvement Review Committee at DHHA.

## Results

OASIS^©^ was implemented across 11 clinics involving 195 clinicians and 29,186 patient encounters. Five rounds of reports were issued (Supplementary Table 1). Adaptations were necessary for implementation (Table 1), most of which required data analytic expertise. Adaptations included the inability to automate OASIS^©^ in organizations that did not utilize an R server but operated R on individual computers. In this case, the program was re-run each month (estimated time 10 minutes). Second, 1 site had difficulty obtaining accurate email addresses from the EHR. In this case, a list of addresses needed to be generated manually. Finally, clinicians requested changes to the report formatting to improve readability (Supplement). The program had high fidelity and significantly improved antibiotic prescribing at CHCO while maintaining good baseline practices at DHHA (Supplement).

## Discussion

We successfully utilized OASIS^©^ to distribute recurrent individualized audit and feedback reports for clinicians. The program demanded adjustments (8 at each site, with an additional 3 at CHCO), with an initial setup time ranging from 1 to 6 hours. Successful integration relied on data analytical skills for code changes in the programming language and analyst access to EHR data. Findings suggest that OASIS^©^ could present a cost-effective approach to antimicrobial stewardship for health systems with adequate data analytics expertise.

Having a team member skilled in informatics and/or coding was essential. We found that the RStudio packages needed to run reports, while free, may be unfamiliar to some analysts. Notably, the code effectively abstracted accurate data for diagnoses and antibiotics and did not require modification despite participating organizations having substantially different Epic^®^ builds.

Although we anticipated the need for adaptations to the code, we were surprised by the clinician and health-system-level adaptations that were required. Institutions had differing security requirements and definitions of Health Insurance Portability and Accountability Act (HIPAA) compliance. For one organization, reports could not depict data for prescribers with fewer than 10 encounters, and some clinicians had their institutional emails forwarded to a personal address and therefore could not receive reports due to HIPAA restrictions. Adaptations were made to ensure that all clinicians ultimately used a compliant email address. In addition, it was noted at CHCO that the read receipt verification prompt was easily bypassed, which led to lower readership because of clinicians not clicking “yes” to having read the report on the pop-up prompt.

This project had several strengths, including the ability to use mixed methods to evaluate adaptations needed for effective implementation of OASIS^©^ and the ability to assess the implementation of 4 relevant metrics. This project also had limitations. We were only able to assess adaptations across 2 health systems that use a similar her; therefore, results may not be generalizable to other systems. Additionally, our evaluation was limited to sites that had some level of data analytic and informatics expertise. Thus, the complexity of implementing OASIS^©^ in sites with limited expertise in using R or accessing the electronic data warehouse may differ. We also could not assess OASIS’s^©^ long-term impact on prescribing due to the short analysis period.

In conclusion, the implementation of OASIS^©^ required contention with system diversity and knowledge gaps in informatics and antibiotic stewardship. Despite these challenges, it proved valuable for monitoring and reporting antimicrobial prescribing. OASIS^©^ could be effectively disseminated to other health systems given the limited time and resources required for adaptations, setup, and monitoring.

## Supporting information

MacBrayne et al. supplementary material MacBrayne et al. supplementary material

## Supplementary material

For supplementary material accompanying this paper visit http://doi.org/10.1017/ash.2024.412. click here to view supplementary material
