# Supplementary material for: Implementation of Outpatient Automated Stewardship Information System (OASIS©) audit and feedback in 2 healthcare systems
Source: Antimicrob Steward Healthc Epidemiol. 2024 Oct 28;4(1):e186. doi: 10.1017/ash.2024.412 (PMC11526177; doi:10.1017/ash.2024.412)
Supplement: MacBrayne et al. supplementary material [file S2732494X24004121sup001.docx]

**S.1. Supplementary methods for secondary outcome measures**

OASIS^©^ uses structured query language (SQL) code in R to access electronic health records from the data warehouse. For Epic, OASIS^©^ requires Clarity access to abstract data but no additional Epic tools.

*Setting and Antimicrobial Stewardship Programs*

OASIS^©^ was implemented at nine family medicine clinics at Denver Health and Hospital Authority (DHHA) and two pediatric clinics at Children’s Hospital Colorado (CHCO). Both DHHA and CHCO have established outpatient antimicrobial stewardship programs led by clinicians. As mentioned, OASIS^©^ reports were sent out on a monthly basis, but no additional reminders were sent out at either location. No additional electronic medical record (EMR) changes were made during the OASIS^©^ pilot.

*Fidelity Monitoring*

We monitored the fidelity of the intervention by documenting read receipts from clinicians who opened the email containing their individualized OASIS^©^ prescribing reports. OASIS^©^ reports were sent via custom OASIS^©^ email addresses at each organization. Attendance at education sessions was captured by meeting minutes and attendance tracking. Meeting criteria for ABP Part 4 MOC credit was tracked in Microsoft Excel for CHCO clinicians.

*Time to Set Up, Barriers, and Facilitators*

Qualitative interviews with staff involved in OASIS^©^ implementation at each organization were conducted to understand the adaptations needed, time required to implement and sustain OASIS^©^, and future modifications that may be helpful to other health systems. Interviews were done virtually using Webex. All interviews were recorded, and results were transcribed. Immersion crystallization was used to identify themes^1^.

*Changes in Antibiotic Prescribing*

Antibiotic utilization for acute respiratory conditions was calculated using an adapted International Classification of Diseases (ICD) code set^2^ that aligned with the Healthcare Effectiveness Data and Information Sets (HEDIS) guide recommendations for the Antibiotic Prescribing for Respiratory Conditions (AXR) measure^3,4^. Unnecessary antibiotic prescribing was measured as the percentage of antibiotics prescribed for respiratory diagnoses for which antibiotics are never indicated, referred to as “Tier 3” in adapted CDC tiering recommendations^5,6^. AOM diagnoses were also identified by ICD codes^7^. First-line antibiotic therapy for AOM was measured as the percentage of antibiotics prescribed for AOM that were amoxicillin^8^. A five-day duration of therapy for AOM was measured as the percentage five-day prescriptions among all antibiotics prescribed for AOM in patients ≥two years old^9^. Encounters where azithromycin or ceftriaxone were prescribed or administered were excluded from the duration of therapy analysis due to having more defined durations of therapy at 5-days and 1-3-days, respectively.

OASIS^©^ clinician prescribing reports comparing individual prescribers to their peers and showing changes in prescribing over time (**Supplemental Figure 1**) were emailed to clinicians who practiced in participating clinics. Education with practical tips to improve prescribing was incorporated into the reports.

We conducted exploratory analyses to determine how the implementation of OASIS^©^ affected antibiotic prescribing. For all metrics, prescribing data from October 2021-October 2023 was electronically abstracted. Data was validated by conducting manual chart review of ten encounters for each data set to assure accuracy of diagnoses and prescriptions. For each metric, prescribing rates pre- and post-OASIS^©^ implementation were compared, as well as their absolute differences, using Pearson’s chi-squared tests. Given our objective to evaluate the impact of OASIS^©^ and the exploratory nature of our analysis, we employed analytical methods and techniques that align with those used in OASIS^©^ clinician reports. We recognized that the treatment approach may reasonably differ for certain children (such as those with an amoxicillin allergy). Due to the limited number of months within the post-intervention period, we opted for direct comparison between pre- and post-intervention periods rather than alternative quasi-experimental designs. We opted not to use a washout period given the short duration of report distribution (2-3 months for each metric) and prior data showing that most changes in prescribing occur within one month of implementation^9^. We defined a statistically significant change by an alpha of 0.05 using two-tailed tests.

**S.2. Supplementary results for secondary outcome measures**

*Fidelity Monitoring*

Clinician engagement was tracked by attendance at an education session (CHCO only), meeting criteria for American Board of Pediatrics (ABP) Part 4 Maintenance of Credit (MOC) (CHCO only), and monitoring who read OASIS^©^ reports. Attendance at the CHCO education session was 15% (12/78). Eight of 12 (67%) CHCO clinicians met criteria for ABP Part 4 MOC credit. To receive Part 4 MOC credit, clinicians had to attend an education session *and* acknowledge that they read at least one of the reports. In total, 91-100% of clinicians at DHHA and 10-30% at CHCO read the emailed reports (**Supplementary Table 1**).

*Time to Set Up, Barriers, and Facilitators*

Qualitative interviews were conducted with four members of the OASIS^©^ project team, comprised of a clinical PharmD and MD and two informatics specialists. Interviews were conducted by a trained research associate (A.K.). The median length of interviews was 12 minutes (range 8-38 minutes). Most of the quality improvement team had no prior experience in creating and sending audit and feedback reports prior to this project.

Key interview findings included 1) data analytic support for initial setup was necessary and challenging; 2) initial implementation of the reports was the most complex; and 3) OASIS^©^ was beneficial in reducing unnecessary prescribing.

*Theme 1: Data Analytic Support* – Data analysts expressed that time for initial setup ranged from 1-6 hours. After automation, the estimated time to send reports was ten minutes. One member of the OASIS^©^ project team said “I think the only part that gave a little bit of trouble was the one R package, RDCom client, has to be installed using devtools R package. It's not like an R package that was easily installed, I mean, that one is not that bad, but it was, it was more roundabout than the other ones at least. Once we have that figured out, it was easy to set everything else up.” Another OASIS^©^ team member reflected, “I think the biggest hurdle for us initially was getting the data analytics resources that we needed to do this and so, I think for other hospitals moving forward it would be important to secure those resources early.”

*Theme 2: Complexity of Use –* There were mixed feelings regarding the complexity of setup. Two of four (50%) interviewees expressed that OASIS^©^ was simple to set up and operationalize, while two (50%) considered it moderately complex. Interviewees agreed that sending the first report was more complex than subsequent reports. One interviewee said, “the first one happened to be the acute otitis media one and I think some of the challenge, some like kinks had to be worked out just because it was the first one. I don't think it had as much to do with the type of metric, just that it was the first time it was getting started and what would it look like, and, you know, like little things like that, like color, text, like, all the things that, um, we would have had to decide, whether it was acute otitis media versus any other metric.”

*Theme 3: Benefits of OASIS*^©^ – All team members perceived OASIS^©^ to be a valuable tool. They believed it increased awareness of outpatient antimicrobial stewardship and influenced antibiotic prescribing among the prescribers who actively engaged with the technology. Both organizations plan to continue and expand use of the program.

*Changes in Antibiotic Prescribing*

Pre-implementation data was collected from October 2021-November 2022. Post-implementation data was collected from July 2023-October 2023. The intervention took place from December 2022 to June 2023. The evaluation included a total of 14,811 patient encounters at DHHA and 14,375 encounters at CHCO, as shown in **Supplementary Table 2.** Pediatricians, family physicians, and advanced practice clinicians contributed to the care of these patients. There was racial and ethnic diversity among the cohort, with 38% of patients at DHHA and 59% at CHCO identifying as non-White race and 66% of patients at DHHA and 48% at CHCO identifying as Hispanic. Additionally, over 80% of the cohort utilized Medicaid.

Baseline first-line antibiotic prescribing for AOM was high at both DHHA (83.7%) and CHCO (79.1%) and showed no significant change post-intervention (**Supplementary Table 3**). Meanwhile, DHHA maintained its high baseline rate (77.4%) for prescribing 5-day durations of therapy for AOM for children aged two years and older, while CHCO observed a significant increase post-intervention from a low baseline of 21.9% to 43.8% (difference 21.9%, 95% CI 16.8, 27.1; p<0.0001).

At DHHA, antibiotic prescribing for respiratory diagnoses, including instances where antibiotics are never indicated, displayed negligible changes in the pre-and post-intervention periods. In contrast, at CHCO, a significant decrease was observed in overall antibiotic prescribing for respiratory conditions (from 8.9% to 6.1%; difference -2.8, 95% CI -3.8, -1.8; p<0.0001), and for cases where antibiotics are never required (from 4.3% to 2.5%; difference -1.9, 95% CI –2.6, -1.2; p<0.0001 **(Supplementary Table 3)**.

**Supplementary Figure 1**. Example Feedback Report for Respiratory Antibiotics Prescribed for Encounters with Any Respiratory Diagnoses


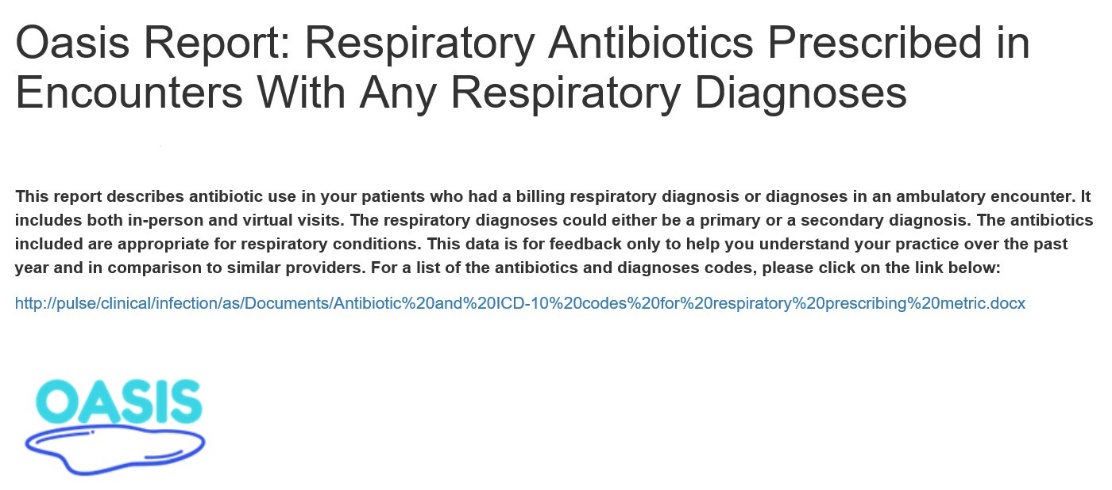


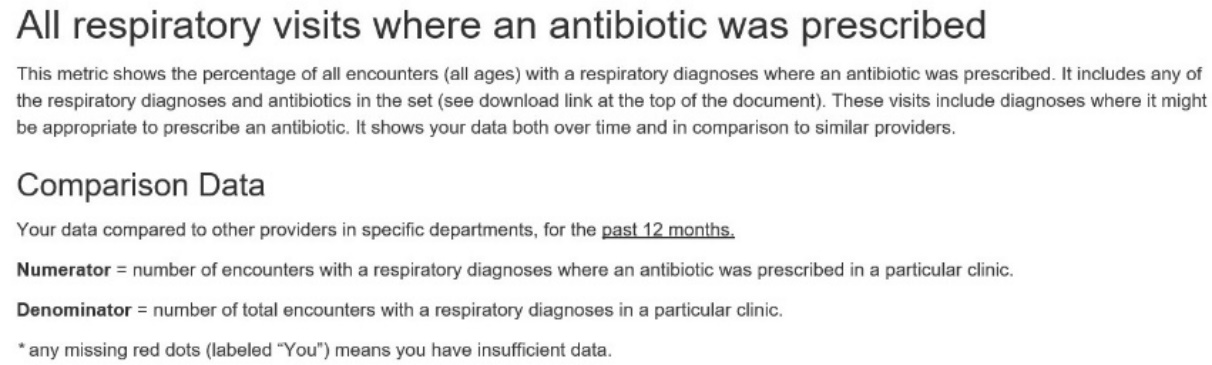


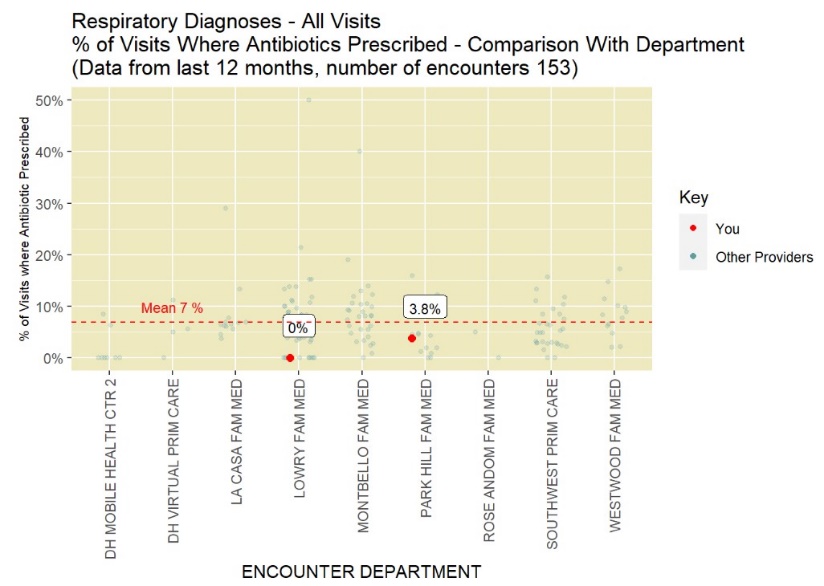


**Supplementary Table 1.** Reporting dates and readership of OASIS reports

| **Date** | **Amoxicillin first line therapy & guideline-concordant durations**  **(N opened/N sent (%))** | **Antibiotic utilization for acute respiratory conditions and for diagnoses where antibiotics are never required**  **(N opened/N sent (%))** |
| --- | --- | --- |
| **Denver Health** | | |
| December 2022 | 38/41 (92.3) | N/A^a^ |
| February 2023 | 33/35 (94.3) | N/A^a^ |
| March 2023 | 21/21 (100) | N/A^a^ |
| April 2023 | N/A^a^ | 71/77 (92.2) |
| June 2023 | N/A^a^ | 52/57 (91.2) |
| **Children’s Hospital Colorado** | | |
| February 2023 | 13/48 (27.1) | N/A^a^ |
| March 2023 | 5/48 (10.4) | N/A^a^ |
| April 2023 | N/A^a^ | 14/46 (30.4) |
| May 2023 | 10/64 (15.6) | N/A^a^ |
| June 2023 | N/A^a^ | 9/41 (21.9) |

^a^Not available. Reports were not sent in these months.

**Supplementary Table 2:** Patient characteristics for eligible encounters for acute otitis media and acute respiratory tract infections for family medicine clinics

|  | Denver Health  N (%)  N= 14,811 | Children’s Hospital Colorado  N (%)  N= 14,375 |
| --- | --- | --- |
| **Health System Characteristics** |  |  |
| Total Participating Clinics | 9 | 2 |
| Family Medicine | 9 | 0 |
| Pediatrics | 0 | 2 |
| **Participating Clinicians Visit Encounters** |  |  |
| Pediatricians | 3,092 (21) | 9,561 (67) |
| Family Medicine Physicians | 5,434 (37) | 0 (0) |
| Advanced Practice Clinicians | 5,196 (35) | 4,814 (33) |
| Other^a^ | 1,089 (7) | 0 (0) |
| **Patient Encounters** |  |  |
| Unique patients | 8,906 | 7,444 |
| Total eligible encounters^b^ | 14,811 | 14,375 |
| Encounters for AXR | 14,811 | 14,375 |
| Encounters for Tier 3 diagnoses | 12,714 | 12399 |
| Encounters for AOM First-line | 770 | 1,478 |
| Encounters for AOM Duration | 508 | 968 |
| **Patient Characteristics Encounter Level** |  |  |
| Age (median (IQR), years) | 8.5 (8.5) | 4.0 (6.2) |
| Male | 8,148 (55) | 7920 (55) |
| R**ace** |  |  |
| African American/Black | 2,639 (18) | 3576 (25) |
| Asian | 866 (6) | 556 (4) |
| White | 9,111 (62) | 5772 (40) |
| Native American | 93 (0.6) | 101 (0.7) |
| Native Hawaiian/Pacific Islander | 79 (0.5) | 61 (0.4) |
| Other Including Multiple Races | 1,900 (13) | 4,178 (29) |
| Declined to Answer/Unknown | 123 (1) | 131 (1) |
| **Ethnicity** |  |  |
| Hispanic | 9,745 (66) | 6888 (48) |
| Non-Hispanic | 4,969 (33) | 7342 (51) |
| Declined to Answer | 97 (1) | 145 (10) |
| **Insurance** |  |  |
| Commercial | 1,027 (7) | 2458 (17) |
| Medicaid | 13,161 (89) | 11720 (82) |
| Other Public | 623 (4) | 197 (1) |
| **Language Preference** |  |  |
| English | 8,182 (44) | 11580 (81) |
| Spanish | 4,828 (33) | 1942 (14) |
| Other | 1,801 (12) | 853 (6) |

^a^Encounters that were not categorized as Pediatrics, Family Medicine or Advanced Practice Clinician.

^b^All patients were included in AXR metric, some patients met eligibility criteria for multiple metrics.

**Supplementary Table 3.** Proportions of antibiotic use pre- and post-intervention.

| Metric | Denver Health^a^ | | | | Children’s Hospital Colorado^b^ | | | |
| --- | --- | --- | --- | --- | --- | --- | --- | --- |
|  | Pre-intervention | Post-intervention | Difference (95% CI) | P-Value | Pre-intervention | Post-intervention | Difference (95% CI) | P-Value |
| First-line therapy for acute otitis media (amoxicillin) | 83.7% (328/392) | 79.1% (299/378) | -4.6% (-10.1- 0.9) | 0.10 | 69.6% (743/1068) | 73.2% (300/410) | 3.6% (-1.5-8.7) | 0.17 |
| 5-day duration of therapy for acute otitis media^c^ | 77.4% (199/257) | 78.9% (194/246) | 1.4% (-5.8-8.7) | 0.70 | 38.0% (260/685) | 63.6% (103/283) | 21.8% (15.9-27.7) | <0.0001 |
| Antibiotic prescribing for respiratory diagnoses^d^ | 7.8% (875/11267) | 8.3% (295/3544) | 0.56% (-0.48-1.6) | 0.28 | 8.9% (981/11057) | 6.1% (201/3318) | -2.8% (-3.8- -1.8) | <0.0001 |
| Antibiotic prescribing for respiratory diagnoses for which antibiotics are **never** indicated^e^ | 2.2%  (217/9696) | 1.8% (54/3018) | -0.45% (-1.0-0.1) | 0.14 | 4.3% (415/9542) | 2.5% (70/2857) | -1.9% (-2.6- - 1.2) | <0.0001 |

^a^The intervention date for Denver Health acute otitis media metrics (first line agent and duration) was 12/2/22 and for overall respiratory prescribing metrics (antibiotic prescribing rate and antibiotics prescribed for Tier 3 diagnoses) was 4/7/23.

^b^The intervention date for Children’s Hospital Colorado acute otitis media metrics (first line agent and duration) was 2/13/23 and for overall respiratory prescribing metrics (antibiotic prescribing rate and antibiotics prescribed for Tier 3 diagnoses) was 4/18/23.

^c^Includes children ≥ 2 years of age prescribed an antibiotic that was not azithromycin or ceftriaxone.

^d^Includes children with Tier 1, 2, and/or 3 respiratory diagnoses

^e^Includes children with a Tier 3 respiratory diagnosis and excludes those who also have a Tier 1 or 2 concurrent diagnosis.

**S.3. Feedback from clinicians**

The following comments were made in response to early iterations of the reports. All comments were addressed throughout the intervention period.

1. “This is challenging to read on mobile.”
2. “I would like a summary statement at the beginning. It’s complicated to know what is important to read. All the info with respiratory visits and comparison data seems like noise.”
3. “These colors are SO difficult to view! Also, the dots are tiny compared to the axis!”

**References**

1. Borkan JM. Immersion-Crystallization: a valuable analytic tool for healthcare research. *Fam Pract*. 2022;39(4):785-789. doi:10.1093/fampra/cmab158

2. Stenehjem E, Wallin A, Willis P, et al. Implementation of an Antibiotic Stewardship Initiative in a Large Urgent Care Network. *JAMA Netw Open*. 2023;6(5):e2313011. doi:10.1001/jamanetworkopen.2023.13011

3. National Committee for Quality Assurance. *HEDIS and Performance Measurement*.; 2021. Accessed September 1, 2023. https://www.ncqa.org/hedis/

4. National Committee for Quality Assurance. *HEDIS Public Comment Overview*.; 2021. Accessed September 1, 2023. https://www.ncqa.org/wp-content/uploads/2021/02/All-Public-Comment-Materials.pdf

5. Bizune D, Tsay S, Palms D, et al. Regional Variation in Outpatient Antibiotic Prescribing for Acute Respiratory Tract Infections in a Commercially Insured Population, United States, 2017. *Open Forum Infect Dis*. 2023;10(2):ofac584. doi:10.1093/ofid/ofac584

6. King LM, Tsay SV, Hicks LA, Bizune D, Hersh AL, Fleming-Dutra K. Changes in outpatient antibiotic prescribing for acute respiratory illnesses, 2011 to 2018. *Antimicrob Steward Healthc Epidemiol ASHE*. 2021;1(1):1-8. doi:10.1017/ash.2021.230

7. Frost Labs. OASIS, Stewardship Simplified. Accessed September 1, 2023. www.oasisstewardship.org

8. Lieberthal AS, Carroll AE, Chonmaitree T, et al. The diagnosis and management of acute otitis media. *Pediatrics*. 2013;131(3):e964-999. doi:10.1542/peds.2012-3488

9. Frost HM, Lou Y, Keith A, Byars A, Jenkins TC. Increasing Guideline-Concordant Durations of Antibiotic Therapy for Acute Otitis Media. *J Pediatr*. 2022;240:221-227.e9. doi:10.1016/j.jpeds.2021.07.016
